# Emergency appointments and psychological distress during COVID-19 lockdown among orthodontic patients in Spain

**DOI:** 10.4317/jced.61081

**Published:** 2024-02-01

**Authors:** María Ochagavía-Berasategui, Susana de la Cruz-Vigo, Iván Nieto-Sánchez

**Affiliations:** 1 UAX Centre for Innovation and Advanced Specialities in Dentistry, Dentistry Faculty, Universidad Alfonso X el Sabio, Madrid, Spain

## Abstract

**Background:**

COVID-19 created an unexpected situation for dental patients. However, the impact of the lockdown on orthodontic patients is still unknown. Hence, this study was conducted to analyse the number and type of emergencies, distress and fear of getting infected among brackets and aligners orthodontic patients during the COVID-19 lockdown.

**Material and Methods:**

A total of 324 questionnaires about emergency appointments during the lockdown and Kessler Psychological Distress Scale-K10 were distributed between March and December 2022 among patients who were on active orthodontic treatment during lockdown at the Master of Orthodontics at the University Alfonso X el Sabio during February and December 2022. Chi-square was used to compare groups.

**Results:**

Emergency appointment was needed by 37.78% of patients during lockdown: 73.5% among those with aligners and 45.9% with brackets (*p*<.01). Loose bracket was the most prevalent emergency for bracket patients and lack of aligners among invisible orthodontic patients. Of those, 36.95% in need of orthodontic emergency appointments didn´t attend the clinic for fear of being infected; 21% of patients had high or very high anxiety levels.

**Conclusions:**

Orthodontic patients had moderate levels of anxiety and fear of being infected during lockdown. Of these, 31.78% needed an emergency appointment.

** Key words:**Orthodontics, COVID-19, dental healthcare, dentistry, emergency.

## Introduction

COVID-19 provoked an epidemiological crisis without precedents in public health globally (1,2). Due to the high transmissibility in dental practices, routine visits had to be cancelled and only emergency treatment was allowed (1-4). Orthodontics often requires monthly visits for long periods. That is why the lack of these controls generated an uncertain scenario for both the orthodontist and the patient. Some patients needed emergency appointments, which could generate extra stress for fear of getting infected. Most dental protocols during the lockdown suggested that dental treatment was limited to emergency treatment: trauma, pericoronaritis, dental infections with pain and inflammation, mucosa perforation due to loose wires…delaying the rest of interventions until the stabilization of the health crisis. This minimizes waiting time and reduces interpersonal contact (5). Spain was one of the nations in the world hit hardest by the coronavirus, in terms of both the number of infected and the number of deaths and the collapse of its health resources (6). Although the measures established by the government during the lockdown were vitally necessary to control the spread of the disease, some studies have shown that reduced social contact, as well as cessation of daily routine, is associated with a high rate of psychosocial stress (3,7). In addition, other recent studies will equate the psychological impact of COVID-19 to post-traumatic stress that can lead to catastrophe, with 1 in 5 people experiencing symptoms of anxiety and depression (8). Although there were protocols to follow for dental treatment during lockdown: triage, temperature check, alcoholic gel, correct ventilation and minimizing waiting and treatment times, many patients refused to attend a dental clinic for fear of getting infected (9). The main aims of this study were to compare the psychological distress of patients with fixed bracket orthodontic appliances and patients with invisible orthodontics, to determine which were the most common emergencies for these patients and the problems the emergencies could cause the patients from the point of view of pain and psychological discomfort and to analyse whether the patients were afraid of contagion for the simple fact of going to the clinic when dealing with their emergencies.

## Material and Methods

The study complied with Helsinki Declaration (10) and was approved by the ethics committee of the University (Resolution 2022_2/120). It was carried out between February and December 2022. All the participants of this study were patients treated by postgraduate orthodontic students at Alfonso X el Sabio University. Assuming an alfa error of 0.05 and a beta error of 0.2 in a bilateral contrast, 317 subjects were needed so that the sample is representative. A total of 324 subjects were surveyed, taking into account missing or incomplete data. Questionnaires with sociodemographic questions, number and type of emergency visits and the Kessler Psychological Distress Scale-K10 (11) were given in paper sheets to participants to complete during the visits after the lockdown, once they were informed about the characteristics of the study and signed the informed consent. This study included patients with fixed multibracket orthodontics or invisible orthodontic patients with transparent aligners, over 18 years of age, both men and women who were treated orthodontically during March and June 2020 (a temporary period in which the Dental Center for Innovation and Advanced Specialties of the Alfonso X el Sabio University remained closed due to the confinement measures imposed by the Spanish government). The cognitive and linguistic capacity of the participants and their competence to understand the characteristics of the study were also necessary to include them in the study. Patients who had completed orthodontic treatment before the beginning of the lockdown or who had started orthodontic treatment after the lockdown. At the same time, the participation of those patients who refused to sign the informed consent was also rejected. Syndromic patients were excluded from this study. The Chi-square test of independence was used to compare two categorical variables. The effect size was calculated to express the magnitude of between-group differences or the relationship between variables.

## Results

Data were collected from 324 patients from about 2/3 of this group aged 18–28 years (64.2%; 208 cases); the others were divided into small groups in the rest of the intervals considered. Women (205) were the majority compared to men (119): 63.3% vs. 36.7% and this majority was maintained in each of the age groups. Half of the sample (51.2%) were students; 38.9% were working as employees; and 2.5% were self-employed. The rest were unemployed. The reported data showed that a large majority of these patients wore braces (89.5%) while the rest (10.5%) had aligner treatment.

It has been found (Table 1) that there were no significant differences in sex (*p*>.05), although there were more women wearing aligners than men.

**Table 1:**
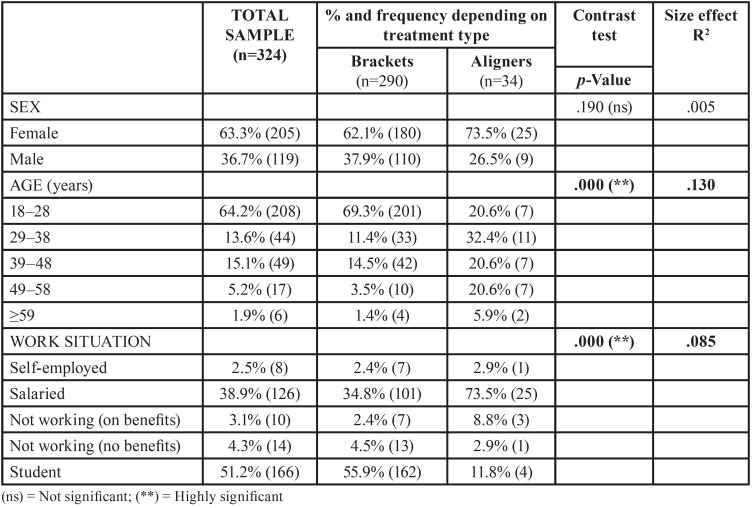
Sociodemographical data: Comparison between treatment groups.

The age distribution showed a clear difference between both groups (*p*<.001) along with an effect size of already large magnitude (.130). The data revealed that the group with braces were younger people between 18 and 28 years (69.3%), while the group with invisible orthodontics the age group was more distributed in several intervals between 18 and 58 years, being in the range of 29-38 years the one of maximum frequency (32.4%).

In the employment situation, there was a high statistical significance (*p*<.001) with a somewhat smaller effect size but still moderate intensity (085). The data indicated that the difference is explained because there were more salaried workers in the group with invisible orthodontics (73.5% vs. 34.8%), while in the group with brackets there were more students (55.9% vs. 11.8%).

**Figure 1:**
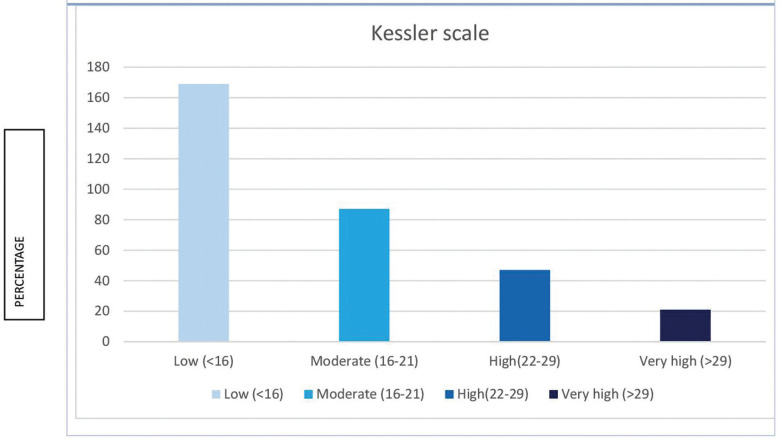
Kessler Psychological Distress Scale (K10) (N=324).

All participants completed the K10 (11), which showed that over 20% of participants had high or very high levels of distress (Fig. 1). There were no differences among brackets or aligner patients (Table 2).

**Table 2:**
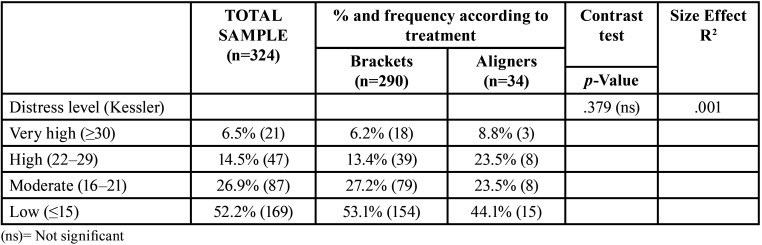
Distress level (Psychological affectation): Comparison between treatment groups.

**Figure 2:**
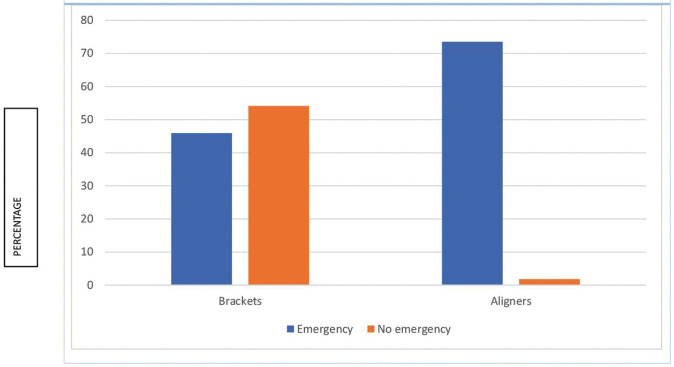
Emergency according to treatment type (N=324).

A total of 48.8% of patients had an emergency that made it necessary to attend the dental clinic. The difference is seen in Figure 2 and clearly shows there were more emergency visits among fixed bracket patients than among aligner patients (*p*>.05).

Table 3 represents the types of urgency recorded. As can be seen, three items stand out as having maximum incidence: having loose brackets (99 cases), having pain (73 cases) and having a loose wire (69 cases). The rest of the problems appear less frequently. In the Table, it can be seen, for example, that the problem of maximum incidence occurs in 63.1% of those who go to the emergency room and in 30.6% of the total sample under study. And so, we can read the rest. The same patient has been able to refer to more than one type of emergency.

**Table 3:**
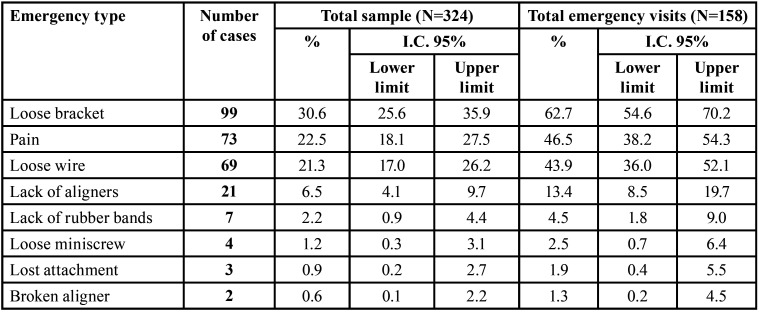
Types of registered emergency visits.

**Figure 3:**
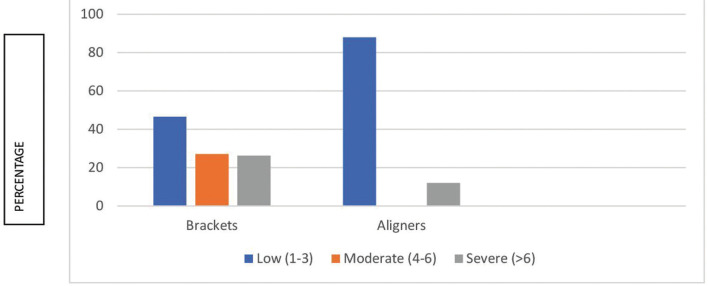
Intensity of pain of those who went to the emergency appointment, according to treatment type (N=158).

**Figure 4:**
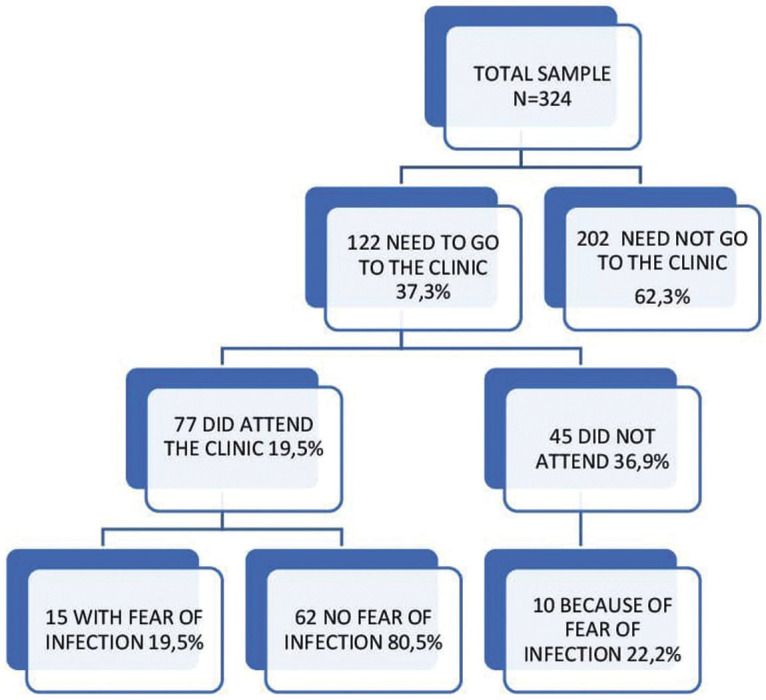
Flow chart regarding fear of being infected.

Table 3 summarizes the differences between both groups regarding the type of emergency experienced. Figure 3 also shows differences between groups regarding the level of pain, which is much higher among bracket patients (*p*<.001). Nevertheless, Table 4 shows that there were no differences between groups in ‘the need to go to the clinic’. Figure 4 shows fear of infection is around 20% in both patients who attended the clinic and those who didn´t even need to.

**Table 4:**
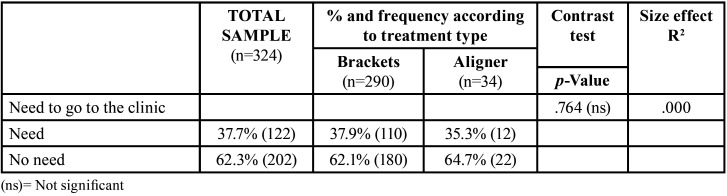
Need to visit the clinic, according to treatment type.

## Discussion

Our study revealed that 21% of patients had psychological distress associated with orthodontic treatment. This agrees with the study by Arqub *et al*. (12) in the United States, who found a low level of stress among orthodontic patients with a K10 score of 13.16±6.63. Xiong *et al*. (13) found that 38% of surveyed patients had mental distress associated with orthodontic treatment, which is slightly higher than the 1/3 of patients showing this distress found by Meriç *et al*. (14). The reason for this difference may be the fact that the questionnaires were distributed in different moments of the pandemic. Salari *et al*. (15) showed in a meta-analysis that levels of anxiety, stress and depression during the lockdown in the general population were around 30%, slightly higher than in our orthodontic sample. Gao *et al*. (16) found higher levels of anxiety among fixed appliance patients compared to aligner patients, which is not in line with our findings. In fact, exposure to mass media has been linked to higher levels of distress and anxiety (17). Gou *et al*. (18) and Cotrin *et al*. (19) found far more emergency among fixed appliances patients, just as we did. Also, the mucosa was damaged because of loose brackets; arch rolling and pain were the most prevalent emergencies in the fixed brackets group in both our sample and other studies (15,18,20-23). Nevertheless, Jones *et al*. (23) found that only 13% of the emergency visits were caused by displaced arches. Nevertheless, aligner patients demanded thrice as many emergency appointments, although only 2% experienced pain, but demanded more aligners, because their main worry was a longer treatment time (15,22). Sometimes the aligners were given to patients, but many authors indicated that there was a shortage of aligners because they were not sent from the manufacturing companies (15,22,23). Only Xiong *et al*. (13) pointed out that aligner patients were less worried than fixed appliances patients because they could at least advance with their treatment changing aligners. Aligner treatment may need auxiliary devices such as miniscrews, buttons, attachments, and rubber bands to make certain movements. These can also be the cause for an emergency appointment (24) and Cotrin *et al*. (19) found that attachment loss was only a minor problem (8.7% of emergency visits), whereas Gou *et al*. (18) found that lost attachments accounted for 32.5% of emergency visits. The loss of rubber bands accounted for 17.6% of emergency appointments in the study of Shenoi *et al*. (25), which is much higher than in our group (5.3%). Although Kaur *et al*. (26) affirmed that aligner treatment seemed to be more adequate for infection control and avoiding transmission because of shorter appointments, fewer appointments, more virtual control, better plaque control and less emergencies were needed, no clear evidence of this affirmation has been published. Popat *et al*. (27) found that emergency appointments were related to pain. In contrast, our sample showed more emergency appointments among aligner patients, but six patients attended because of pain related to dental movement, five of whom had braces. Higher pain levels were reported among bracket patients (16). Nevertheless, there is still controversy about whether brackets or aligners cause more dental pain regarding tooth movements (25,28). Mendonça *et al*. (29) showed more pain levels among patients with higher levels of anxiety and changes in daily routine among orthodontic patients. Quan *et al*. (30) showed that 33.67% of orthodontic patients had troubles during the lockdown, quite close to 37.7% of our patients. Most authors concluded that patients requiring emergency visits showed higher levels of distress (13,19,30). In our sample between 19.5% and 22.2% of patients showed fear of infection of COVID-19, much lower than 55% found by Quan *et al*. (30). It seems that extending treatment time was one of the causes of anxiety for many patients (13,14,19). This study has some limitations: most patients were female and there were more patients with brackets than aligners, so differences between groups may not be representative. There may be a social desirability bias, because some patients may not admit that they did not attend the clinic for fear of being infected but refuse to say so once the lockdown is over and the pandemic situation has improved.

## Conclusions

Patients with invisible orthodontics were less affected by emergencies, lost appointments, and pain, which resulted in better mental health than fixed multibracket appliance patients, who reported higher levels of anxiety and lower well-being associated with orthodontic emergencies.

Mucosa ulcerations and brackets debonding, and rolling of arches were the most common emergencies among fixed multibracket appliance patients, whereas running out of aligners was the most prevalent ‘emergency’ among invisible aligner patients. Fixed orthodontic appliances reported higher pain levels, but invisible aligner patients demanded more ‘urgent’ assistance.

Most patients who required in-person assistance to solve their problems went to the clinic without fear of getting infected; only a small amount of them didn´t turn up to the clinic for fear of getting infected.

## References

[1] Farrokhi F, Mohebbi SZ, Farrokhi F, Khami MR (2021). Impact of COVID-19 on dental education - a scoping review. BMC Med Educ.

[2] Bastani P, Mohammadpour M, Ghanbarzadegan A, Kapellas K, Do LG (2021). Global concerns of dental and oral health workers during COVID-19 outbreak: a scope study on the concerns and the coping strategies. Syst Rev.

[3] Jiménez-Etxebarria E, Iturrioz EB, Jaureguizar J (2021). Impact of the COVID-19 pandemic as perceived by older people in Northern Spain. Psychol Res Behav Manag.

[4] Malekshoar M, Malekshoar M, Javanshir B (2021). Challenges, limitations, and solutions for orthodontists during the coronavirus pandemic: a review. Am J Orthod Dentofacial Orthop.

[5] Siles-Garcia AA, Alzamora-Cepeda AG, Atoche-Socola KJ, Peña-Soto C, Arriola-Guillén LE (2021). Biosafety for dental patients during dentistry care after COVID-19: a review of the literature. Disaster Med Public Health Prep.

[6] Pollán M, Pérez-Gómez B, Pastor-Barriuso R, Oteo J, Hernán MA, Pérez-Olmeda M (2020). Prevalence of SARS-CoV-2 in Spain (ENE-COVID): a nationwide, population-based seroepidemiological study. Lancet.

[7] Campagne DM (2019). Stress and perceived social isolation (loneliness). Arch Gerontol Geriatr.

[8] Ausín B, Muñoz M, Castellanos MA (2017). Loneliness, sociodemographic and mental health variables in Spanish adults over 65 years old. Span J Psychol.

[9] González-Olmo MJ, Delgado-Ramos B, Ortega-Martínez AR, Romero-Maroto M, Carrillo-Díaz M (2022). Fear of COVID-19 in Madrid. Will patients avoid dental care? Int Dent J.

[10] World Medical Association (2013). World Medical Association Declaration of Helsinki: ethical principles for medical research involving human subjects. JAMA.

[11] Kessler RC, Andrews G, Colpe LJ, Hiripi E, Mroczek DK, Normand SL (2002). Short screening scales to monitor population prevalences and trends in non-specific psychological distress. Psychol Med.

[12] Arqub SA, Voldman R, Ahmida A, Kuo CL, Godoy LDC, Nasrawi Y (2021). Patients' perceptions of orthodontic treatment experiences during COVID-19: a cross-sectional study. Prog Orthod.

[13] Xiong X, Wu Y, Fang X, Sun W, Ding Q, Yi Y (2020). Mental distress in orthodontic patients during the coronavirus disease 2019 pandemic. Am J Orthod Dentofacial Orthop.

[14] Meriç P, Naoumova J (2022). Did the coronavirus disease 2019 pandemic affect orthodontic treatment outcomes? A clinical evaluation using the objective grading system and Peer Assessment Rating index. Am J Orthod Dentofacial Orthop.

[15] Salari N, Hosseinian-Far A, Jalali R, Vaisi-Raygani A, Rasoulpoor S, Mohammadi M (2020). Prevalence of stress, anxiety, depression among the general population during the COVID-19 pandemic: a systematic review and meta-analysis. Global Health.

[16] Gao M, Yan X, Zhao R, Shan Y, Chen Y, Jian F (2021). Comparison of pain perception, anxiety, and impacts on oral health-related quality of life between patients receiving clear aligners and fixed appliances during the initial stage of orthodontic treatment. Eur J Orthod.

[17] Liu M, Zhang H, Huang H (2020). Media exposure to COVID-19 information, risk perception, social and geographical proximity, and self-rated anxiety in China. BMC Public Health.

[18] Gou Y, Ungvijanpunya N, Chen L, Zeng Y, Ye H, Cao L (2022). Clear aligner vs fixed self-ligating appliances: orthodontic emergency during the 2020 coronavirus disease 2019 pandemic. Am J Orthod Dentofacial Orthop.

[19] Cotrin P, Peloso RM, Pini NIP, Oliveira RC, de Oliveira RCG, Valarelli FP (2020). Urgencies and emergencies in orthodontics during the coronavirus disease 2019 pandemic: Brazilian orthodontists' experience. Am J Orthod Dentofacial Orthop.

[20] Xiong J, Lipsitz O, Nasri F, Lui LMW, Gill H, Phan L (2020). Impact of COVID-19 pandemic on mental health in the general population: a systematic review. J Affect Disord.

[21] García-Álvarez L, de la Fuente-Tomás L, García-Portilla MP, Sáiz PA, Lacasa CM, Dal Santo F (2020). Early psychological impact of the 2019 coronavirus disease (COVID-19) pandemic and lockdown in a large Spanish sample. J Glob Health.

[22] Martinez-Garcia M, Rabasa A, Barber X, Polotskaya K, Roomp K, Oliver N (2021). Key factors affecting people's unwillingness to be confined during the COVID-19 pandemic in Spain: a large-scale population study. Sci Rep.

[23] Buffel V, van de Straat V, Bracke P (2015). Employment status and mental health care use in times of economic contraction: a repeated cross-sectional study in Europe, using a three-level model. Int J Equity Health.

[24] Jones K, Popat H, Johnson IG (2016). Dental students' experiences of treating orthodontic emergencies - a qualitative assessment of student reflections. Eur J Dent Educ.

[25] Shenoi SB, Deshpande S, Jatti R (2020). Impact of COVID-19 Lockdown on patients undergoing orthodontic treatment: A Questionnaire Study. J Indian Orthod Soc.

[26] Kaur H, Kochhar AS, Gupta H, Singh G, Kubavat A (2020). Appropriate orthodontic appliances during the COVID-19 pandemic: a scoping review. J Oral Biol Craniofac Res.

[27] Popat H, Thomas K, Farnell DJ (2016). Management of orthodontic emergencies in primary care - self-reported confidence of general dental practitioners. Br Dent J.

[28] Sánchez-Rodríguez E, Ferreira-Valente A, Pimenta F, Ciaramella A, Miró J (2022). Mental, physical and socio-economic status of adults living in Spain during the late stages of the state of emergency caused by COVID-19. Int J Environ Res Public Health.

[29] Mendonça DL, Almeida-Pedrin RR, Pereira NC, Oltramari PVP, Fernandes TMF, Conti ACCF (2020). The influence of text messages and anxiety on pain perception and its impact on orthodontic patients routine. Dental Press J Orthod.

[30] Quan S, Guo Y, Zhou J, Zhang G, Xing K, Mei H (2021). Orthodontic emergencies and mental state of Chinese orthodontic patients during the COVID-19 pandemic. BMC Oral Health.

